# Cross-Polarization of Insensitive Nuclei from Water Protons for Detection of Protein–Ligand Binding

**DOI:** 10.1021/jacs.4c08241

**Published:** 2024-09-03

**Authors:** Nirmalya Pradhan, Christian Hilty

**Affiliations:** Chemistry Department, Texas A&M University, 3255 TAMU, College Station, Texas 77843, United States

## Abstract

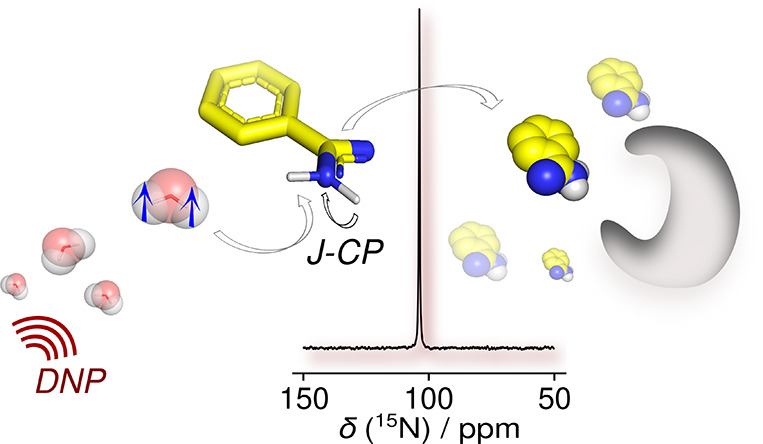

Hyperpolarization derived from water protons enhances the NMR signal of ^15^N nuclei in a small molecule, enabling the sensitive detection of a protein–ligand interaction. The water hyperpolarized by dissolution dynamic nuclear polarization (D-DNP) acts as a universal signal enhancement agent. The ^15^N signal of benzamidine was increased by 1480-fold through continuous polarization transfer by *J*-coupling-mediated cross-polarization (*J*-CP) via the exchangeable protons. The signal enhancement factor favorably compares to factors of 110- or 17-fold using non-CP-based polarization transfer mechanisms. The hyperpolarization enabled detection of the binding of benzamidine to the target protein trypsin with a single-scan measurement of ^15^N *R*_2_ relaxation. *J*-CP provides an efficient polarization mechanism for ^15^N or other low-frequency nuclei near an exchangeable proton. The hyperpolarization transfer sustained within the relaxation time limit of water protons additionally can be applied for the study of macromolecular structure and biological processes.

The amplification of signal from hyperpolarization enables NMR spectroscopy at physiologically relevant concentrations and improves the time resolution for the study of biochemical processes.^1−3^ Dissolution dynamic nuclear polarization (D-DNP)^4^ increases signals of low-frequency nuclei such as ^13^C and ^15^N by 3–4 orders of magnitude. Spectra measured *in vitro* or *in cellulo* reveal biosynthetic and metabolic pathways,^5^ reaction kinetics and mechanisms,^6^ protein folding,^7^ and other processes on the subsecond time scale.^8^ At equilibrium, hyperpolarization facilitates the measurement of macromolecular interactions, binding affinity,^9^ or the structures of binding interfaces and pockets.^10^

Although DNP readily polarizes the nuclear spins of most molecules in frozen solids, the decay of the hyperpolarization is accelerated after transfer to the liquid state. In aqueous solution, the available time and signal levels may be substantially extended by first hyperpolarizing water protons. Polarization from this reservoir can transfer repeatedly to the molecule of interest through proton exchange and dipolar interactions.^11^ Proton signals become enhanced for a period governed by the *T*_1_ relaxation time of water protons.^12^ This technique, also termed HyperW, removes the obstacle of fast relaxation for *ex situ* polarized bio-macromolecular spins, while retaining the physiological environment of the molecules to support native conformations.^13,14^ It holds promise to broaden the applications of NMR for structural biology, biomolecular dynamics,^15^ protein–ligand interactions, and others.^16^

We demonstrate the substantial potential that water hyperpolarization holds for the signal enhancement of a low-frequency nucleus, ^15^N. We then apply this technique for the measurement of the binding of benzamidine, a reversible inhibitor of trypsin and trypsin-like proteases. While the ability to hyperpolarize proton spins of a target molecule is improved by exchange with water protons, a short complex lifetime reduces the amount of polarization transferred to neighboring spins. This limitation is overcome by *J*-coupling-mediated cross-polarization (*J*-CP).

First, one of the nitrogen atoms in benzamidine was enriched with ^15^N (Scheme S1 and Figures S1–S5).^17^ Hyperpolarization of water was generated using DNP in a frozen solid at 1.4 K. Subsequent dissolution in D_2_O and rapid injection into a 400 MHz NMR spectrometer resulted in a water signal that was 40 ± 2-fold larger than the signal from a non-hyperpolarized pure water sample, despite a 1:26 dilution with deuterium.^18^ The signal enhancement of water protons from DNP was ∼2400-fold, resulting in an ∼1100-fold effective enhancement after introduction of residual protons of the dissolution solvent. *J*-CP transfer of this polarization under RF irradiation resulted in a ^15^N signal of benzamidine that was 757 ± 58-fold enhanced compared with non-hyperpolarized *J*-CP (Figure 1). Hyperpolarized *J*-CP also exhibited 1480 ± 110-fold ^15^N signal enhancement in contrast with non-hyperpolarized direct ^15^N measurements.

**Figure 1:**
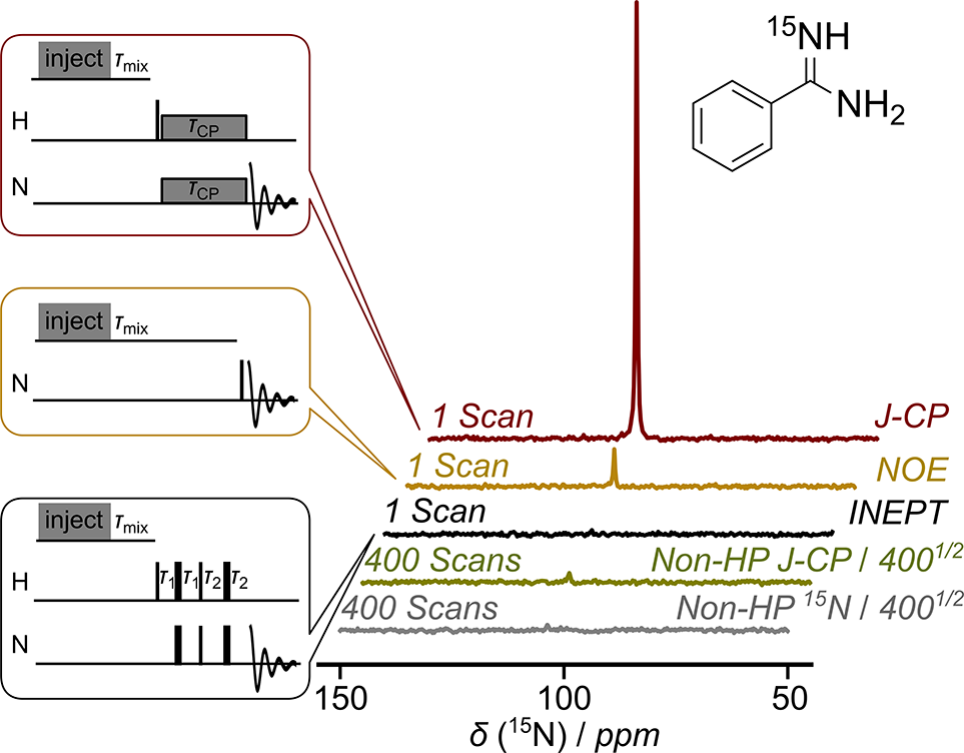
Polarization transfer from hyperpolarized water to 9.1 mM, 9.6 mM, and 9.1 mM benzamidine, employing *J*-CP, NOE, and INEPT, at pH 7.25. The injection of hyperpolarized water was followed by τ_mix_ = 500 ms, 15 s, and 500 ms, respectively. *J*-CP used τ_CP_ = 160 ms, *γB*_1_ = 3125 Hz, ω_N_ = 105 ppm, and ω_H_ = 8 ppm. Refocused INEPT included τ_1_ = 1/(4*J*_HN_), τ_2_ = 1/(6*J*_HN_), and *J*_HN_ = 92 Hz. Non-hyperpolarized experiments were performed with a sample from a previous hyperpolarized experiment containing 9.08 mM benzamidine, in 400 scans. The vertical scale is reduced by 400^1/2^ to match noise levels.

Continuous transfer of water hyperpolarization can also occur through the NOE.^12,19^ A ^15^N signal enhancement of 110 ± 18-fold was observed after a contact time of 15 s. A refocused “insensitive nuclei enhanced by polarization transfer” (INEPT) scheme increased the ^15^N magnetization through the *J*-coupling merely by a factor of 17 ± 6 (Figure 1). As the NOE in small molecules is inefficient due to rapid motions and the otherwise preferred INEPT technique provided even less signal, neither of these options is ideal. The inefficiency of INEPT is explained by the fast-exchange kinetics of the water protons with the amidine protons. The exchange rate, *k*_ex_, due to the hydroxide base catalyzed exchange mechanism at the physiological pH of 7.25 is estimated as 1700 s^–1^ (Figure S7).^20,21^ This rate is much higher than *J*_HN_ = 92 Hz, which severely limits the INEPT polarization transfer. The limitation is overcome with *J*-CP, an effective method for sensitivity enhancement of low-frequency nuclei in solution.^22,23^ With proton exchange, the *J*-CP polarization transfer can occur continuously during the CP mixing time. The *J*-CP with Hartmann–Hahn matched spin-locking fields on ^1^H and ^15^N has provided improved polarization transfer under fast solvent exchange conditions in proteins without hyperpolarization.^24,25^ The data in Figure 1 demonstrate that HyperW with *J*-CP is an efficient approach for biomolecular polarization.

Because HyperW experiments generally dissolve hyperpolarized water in D_2_O, we explored the CP efficiency with variable water percentages (Figure S8). In these experiments performed without hyperpolarization, the maximum signal reached within 160 ms of CP buildup time is nearly independent of the water proton content. However, with higher deuterium enrichment, a shift in the maximum toward a longer CP time is observed. Concomitantly, upon lowering the water proton content from 90% to 10%, the initial slope of the buildup curve was reduced by 40%. This difference is explained by the fact that the polarization transfer originates primarily from the proton spins. Additionally, deuteration decreases the spin–lattice relaxation rates for water protons from 0.28 s^–1^ to 0.09 s^–1^ (Figure S9a–c). Under fast exchange, the ^15^N magnetization achieved in a given CP time depends on ^15^N and water proton *T*_1_ and not significantly on the *T*_1_ of the ^15^N-bound exchangeable proton.^25^ The reduced relaxation in the deuterated solutions, combined with the slower polarization transfer, explains the aforementioned shift of the signal maximum to a longer CP time.

The opposing effects on the relaxation and polarization transfer rates also equalize the maximum achievable ^15^N signal at different ratios of water and deuterium seen in Figure S8. The transferable signal in the *J*-CP experiment apparently depends primarily on the water proton polarization, even if the magnitude of the water signal is reduced with a lower proton concentration. In contrast, HyperW experiments that observe signals from exchangeable protons directly yield a signal proportional to the product of water proton concentration and polarization. By retaining its effectiveness at the lowered proton concentration, the *J*-CP experiment is ideally compatible with D-DNP. Due to the dilution of the hyperpolarized water with D_2_O during the dissolution, *J*-CP supports a higher polarization of the target nucleus than HyperW alone.

Figure 2 compares the signal buildup from hyperpolarized water to the ^15^N of benzamidine due to *J*-CP or NOE. In *J*-CP, the maximum buildup is beyond the longest mixing time of 160 ms. This result was expected, since hyperpolarized solutions contain only 3.9 ± 0.9% of water due to dilution in D_2_O. The water *T*_1_ was prolonged to 5.1 s, despite a relaxation contribution from 0.45 mM TEMPOL in the sample after dissolution (Figure S9e,f).

**Figure 2:**
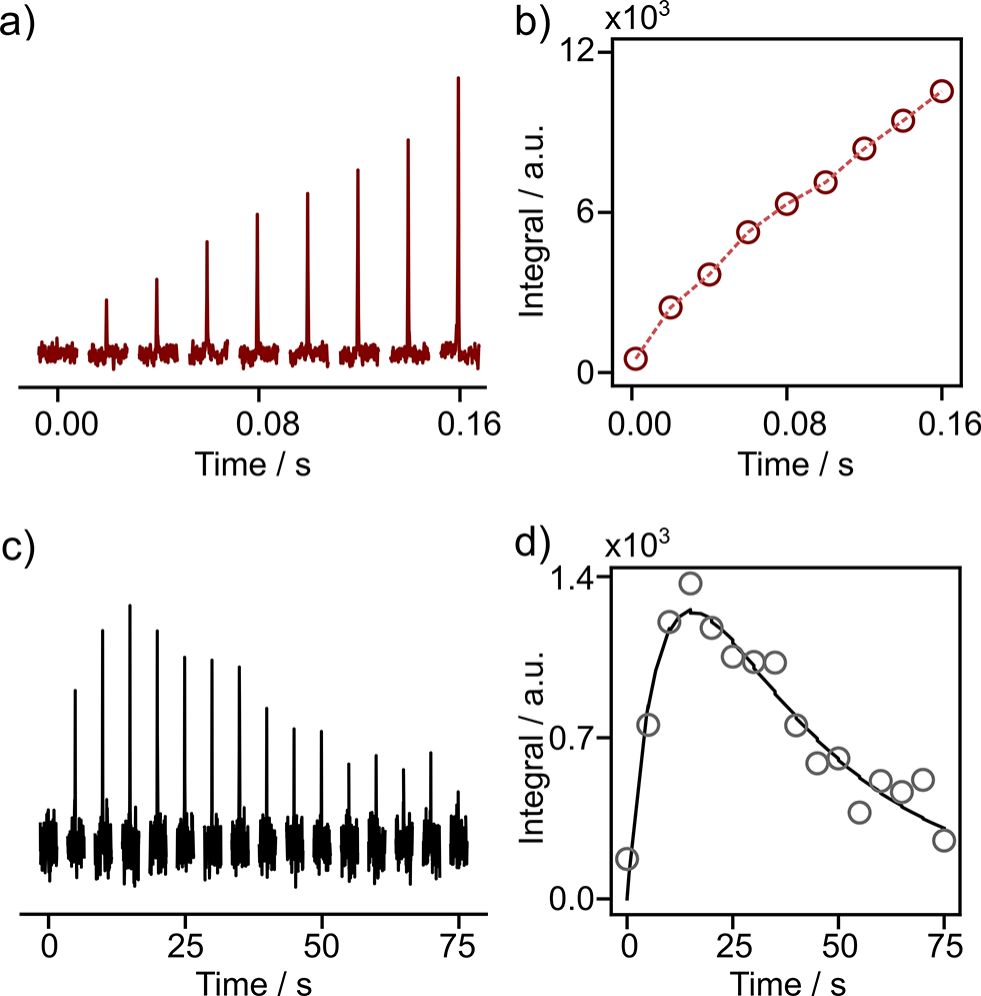
(a) ^15^N NMR signals of 1.23 ± 0.11 mM benzamidine transferred from hyperpolarized water using *J*-CP with different CP times. (b) Buildup curve of ^15^N magnetization achieved in (a). (c) Time-dependent ^15^N NMR signals measured from 14.6 mM benzamidine following NOE transfer of polarization from hyperpolarized water. Spectra were acquired with 9° pulses at intervals of 5 s. (d) Fitted curve of integrals from (c), resulting in *R*_1_^N^ = 0.13 s^–1^, *R*_1_^H^ = 0.03 s^–1^, and *k*_H→N_ = 1.41 s^–1^. In (a) and (b), τ_mix_ = 500 ms. Integrals were normalized with the benzamidine concentration and for the flip-angle of the pulse.^12,19^

The *J*-CP efficiency leading to the observed buildup curve depends on the amidine proton exchange rate. The ^15^N signal integrals in a *J*-CP experiment with benzamidine decrease at high pH, where the rate is increased (Figure S10). A reduction of the *J*-CP efficiency with an exchange rate that is significantly larger than *J*_HN_ has also been seen in ref (25). Nevertheless, the efficiency of *J*-CP sharply surpasses that of INEPT for a fast-exchanging proton.

The *J*-CP polarization transfer also depends on experimental parameters, including the RF field strength γ*B*_1_, frequency offsets, and quality of radio frequency power matching (Figure S11). The DIPSI sequence is used in the experiment to improve the offset dependence.^26,27^ The γ*B*_1_ = 3125 Hz of this sequence is sufficient to cover the line width of the amidine signal at pH 7.25 and at least partially spin-lock the signal of water, while preventing excessive RF power deposition.

The analysis of the NOE buildup and decay curve shows a maximum ^15^N polarization at only 15 s (Figure 2d). In addition, the starting slope of the CP buildup curve was 440 times larger than that of the NOE (Figure 2c). Different from the coherent magnetization transfer in *J*-CP, the NOE buildup depends on relaxation rates of amidine ^15^N and ^1^H, as well as hyperpolarized water.^12^ The curve reflects the longitudinal relaxation rate of 0.167 ± 0.061 s^–1^ and the overall polarization transfer rate from water to ^15^N due to exchange and NOE processes, *k*_H→N_ = 1.57 ± 0.13 s^–1^. Since the amidine ^1^H is in fast exchange with hyperpolarized water at a pH of 7.25, the intramolecular polarization transfer rate is the rate-limiting step. As a result, the NOE enhanced ^15^N polarization is approximately 13 times less than that in the *J-*CP experiment.

The signal-to-noise ratio (SNR) in the *J*-CP experiment in Figure 1 is ≥550. The detection limit corresponding to an SNR of 3 would be reached with ∼50 μM substrate (SI Section 7). This substrate concentration enables the study of biological systems at or near physiological conditions, even while observing the otherwise insensitive ^15^N nucleus. It would further be possible to observe ∼12.5 mM of unenriched substrate at the 0.4% natural abundance of ^15^N with an SNR of 3.

In the following, enhanced ^15^N magnetization from *J*-CP polarization transfer was used to measure the interaction of benzamidine with the protein trypsin. The *R*_2_ relaxation rates of the polarized ^15^N spin in benzamidine were determined by a single-scan Carr–Purcell–Meiboom–Gill (CPMG) pulse sequence incorporating *J*-CP transfer (Figure S6). The relaxation rate increased from *R*_2_ = 31.3 ± 2.5 s^–1^ for benzamidine alone to 41.1 ± 4.0 s^–1^ in the presence of trypsin protein (Figure 3 and Table S2; average of three measurements each with error ranges propagated from fit). The increased *R*_2_ relaxation rate indicates binding of benzamidine with trypsin.

**Figure 3:**
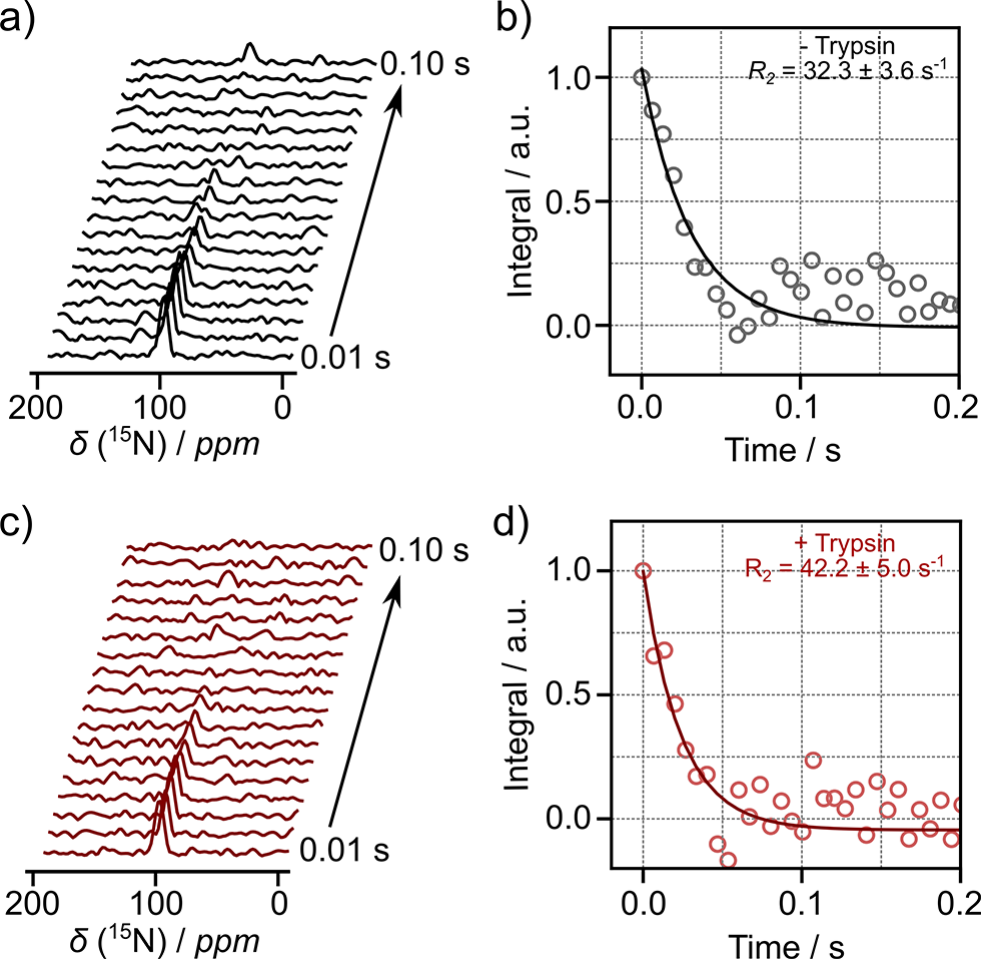
(a) ^15^N NMR spectra of 0.95 mM polarized benzamidine from the echoes in a single-scan CPMG experiment following *J*-CP. (b) Fitted exponential curve measuring *R*_2_ from (a). (c) ^15^N spectra of 0.88 mM polarized benzamidine with 50 μM trypsin. (d) Fitted curve from (c). All experiments were performed with hyperpolarized water at pH 7.25 ± 0.04, an echo time of 6.7 ms, and τ_mix_ = 500 ms. The integrated data points were normalized to the first point.

The *R*_2_ relaxation rate of the free ligand is relatively fast, likely due to exchange effects. These effects may among others include the influence of H/H and H/D exchange, which can result in the loss of antiphase coherences that appear between refocusing pulses.^28^ When the protein is bound, the exchange contribution may be reduced, but the increased rotational correlation time of the protein–ligand complex substantially increases *R*_2_. In Figure 3, the change in the relaxation rate upon binding to the protein was observable, despite the relatively fast relaxation rate of free benzamidine.

The data in Figure 3 emphasize the possibility of ^15^N NMR to prove a biological interaction in a single experiment. The binding affinity may further be determined by measuring *R*_2_ at different concentrations of the ligand or the protein, or using competitive binding experiments.^29,30^ Practically, a non-hyperpolarized ^15^N relaxation experiment will require ≥560,000 signal averages for gaining the same signal-to-noise ratio as in the hyperpolarized *J*-CP experiment with the signal enhancement of ≥750. In contrast, the single scan experiment using hyperpolarized water was performed in 40 min, including the water polarization time.

Hyperpolarization of ^15^N through HyperW opens new applications benefiting from well-dispersed and background-free ^15^N NMR signals. These include the screening of ligand interactions in drug development and the study of receptors and other targets that are important for cellular functions. Nitrogen nuclei with exchangeable protons are ubiquitous in biological macromolecules, including proteins and nucleic acids. Nitrogen is also part of small molecules such as adenosine diphosphate, adenosine triphosphate, cyclic guanosine monophosphate, biotin, dopamine, and *gamma*-aminobutyric acid, which are all involved in cell signaling. These molecules represent a large pool of substrates for NMR-based cell signaling studies. In proteins, polarized ^15^N relaxation experiments can provide insight into local backbone dynamics and transient protein–protein interactions. Hyperpolarized ^15^N relaxation dispersion may further identify binding epitopes of ligands with macromolecules.^31^^15^N polarization may also be adopted to perform in-cell NMR studies that provide structural and dynamic information about biological molecules and processes under physiological conditions.^32^ The ^15^N polarization may further be exploited to detect biomarkers for applications in metabolomic research.^33,34^

In summary, the ^15^N hyperpolarization of benzamidine using hyperpolarized water and *J*-CP was demonstrated. The transferred polarization outweighed both INEPT- and NOE-based pulse sequences in the molecule containing a fast-exchanging proton. The enhanced signals were used to measure the interaction of the molecule with trypsin as a target protein. The use of hyperpolarized water for the polarization of low-γ nuclei is applicable to various biological small molecules and macromolecules, for measuring protein–ligand binding, protein dynamics, protein–protein interactions, and others, in a native environment and short time frame.

## Supporting Information Available

Synthesis of ^15^N-benzamidine, experimental methods, exchange rate calculation, *J*-CP buildup curve, water *T*_1_ relaxation experiments, measurement of *J*-CP efficiency, ^15^N signal-to-noise ratio, NOE data, and protein interaction data (PDF)
