# Supplementary material for: Cross-Polarization of Insensitive Nuclei from Water Protons for Detection of Protein–Ligand Binding
Source: J Am Chem Soc. 2024 Sep 3;146(36):24754–8. doi: 10.1021/jacs.4c08241 (PMC11403598; doi:10.1021/jacs.4c08241)
Supplement: Supplementary file 1 — ja4c08241_si_001.pdf [file ja4c08241_si_001.pdf]

Supporting Information for

Cross-Polarization of Insensitive Nuclei from Water Protons for Detection of Protein-Ligand Binding

Nirmalya Pradhan and Christian Hilty*

Chemistry Department, Texas A&M University, College Station, TX 77843, USA

* chilty@tamu.edu

Table of Contents

1. Synthesis of ^{15}N-Benzamidine....S2
   1.1. General Information....S2
   1.2. Synthesis Protocols....S2
2. Experimental Methods....S5
   2.1. Sample Preparation....S5
   2.2. Water Hyperpolarization....S5
   2.3. NMR Experiments....S5
      2.3.1. Non-Hyperpolarized ^{15}N NMR Experiments....S5
      2.3.2. Non-Hyperpolarized *J*-CP NMR Experiments....S6
      2.3.3. HyperW NMR Experiments....S6
      2.3.4. Relaxation Experiments with Protein....S7
3. Exchange Rate Calculation....S7
4. *J*-CP Buildup Curve....S8
5. Water T_1 Relaxation Experiments....S8
6. Measurement of *J*-CP Efficiency....S9
   6.1. Effect of Exchange Rates....S9
   6.2. Effect of *J*-CP Parameters....S10
   6.3. Line Shape and Frequency Difference....S11
7. ^{15}N Signal-to-Noise Ratio....S11
8. NOE Data....S11
9. Protein Interaction Data....S12
10. References....S12

1. Synthesis of ^{15}N-Benzamidine

1.1. General Information

All the reagents and solvents were purchased from various commercial sources and used for synthesis without further purification. The synthesized compound was characterized by a Bruker 400 MHz NMR spectrometer (^{1}H at 400 MHz, ^{13}C{^{1}H} at 100 MHz, ^{13}C{^{1}H, ^{15}N} at 100 MHz and ^{15}N at 40 MHz). High-resolution mass spectra were measured by the Laboratory for Biological Mass Spectrometry at Texas A&M University, College Station, TX 77840. Electrospray ionization mass spectrometry (ESI- MS) experiments were performed using a Thermo Scientific Q Exactive Focus.

1.2. Synthesis Protocols

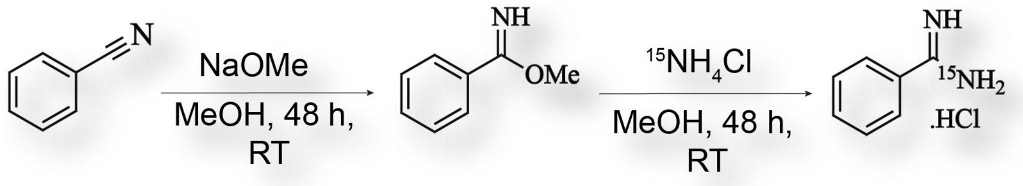

Scheme S1. *Synthesis of ^{15}N benzamidine hydrochloride.*

The ^{15}N enriched benzamidine hydrochloride was synthesized following a reported synthetic procedure with minor modifications.[1] Briefly, to a stirring solution of benzonitrile (0.5 g, 4.85 mmol) in dry methanol, sodium methoxide (27 mg, 0.5 mmol) was added. The resultant mixture was stirred overnight at room temperature. Subsequently, ^{15}N labeled ammonium chloride (518 mg, 9.7 mmol) was added. The reaction solution was then stirred at room temperature for an additional two days. The reaction solution was concentrated under reduced pressure and ethanol was added. The undissolved solid was filtered and the filtrate was again concentrated under reduced pressure. Finally, the crude product was washed with cold ethanol and dried under vacuum, resulting in a white solid with 50% yield (380 mg). The product was analyzed using ^{1}H NMR, ^{13}C NMR, ^{15}N NMR and mass-spectrometry (MS). The chemical shifts were referenced to a standard sample containing sodium trimethylsilylpropanesulfonate (DSS). **^{1}H NMR (400 MHz, 9:1 H_2O/D_2O)** δ(ppm) 7.75 (m, 3H), 7.60 (m, 2H). **^{13}C{^{1}H} NMR (100 MHz, 9:1 H_2O/D_2O)** δ(ppm) 169.6 (m), 136.9, 132.0, 130.5, 130.4. **^{13}C{^{1}H, ^{15}N} NMR (100 MHz, 9:1 H_2O/D_2O)** δ(ppm) 169.6, 136.9, 132.0, 130.5, 130.4. **^{15}N NMR (40 MHz, 9:1 H_2O/D_2O)** δ(ppm) 103.6. HRMS (ESI) calculated for $[C_7H_9N^{15}N]^+$: 122.0731, found: 122.0731.

Proton decoupled ^{13}C NMR spectra of synthesized benzamidine showed a multiplet peak at 169.6 ppm corresponding to the amidine carbon (Fig. S2). The splitting of the ^{13}C signal indicates the coupling to ^{15}N nuclei. On the other hand, both ^{1}H and ^{15}N decoupled ^{13}C spectra showed singlets at the same chemical shift (Fig. S3). ^{13}C {^{1}H, ^{15}N} and HRMS (ESI) analysis confirm the incorporation of ^{15}N in benzamidine.

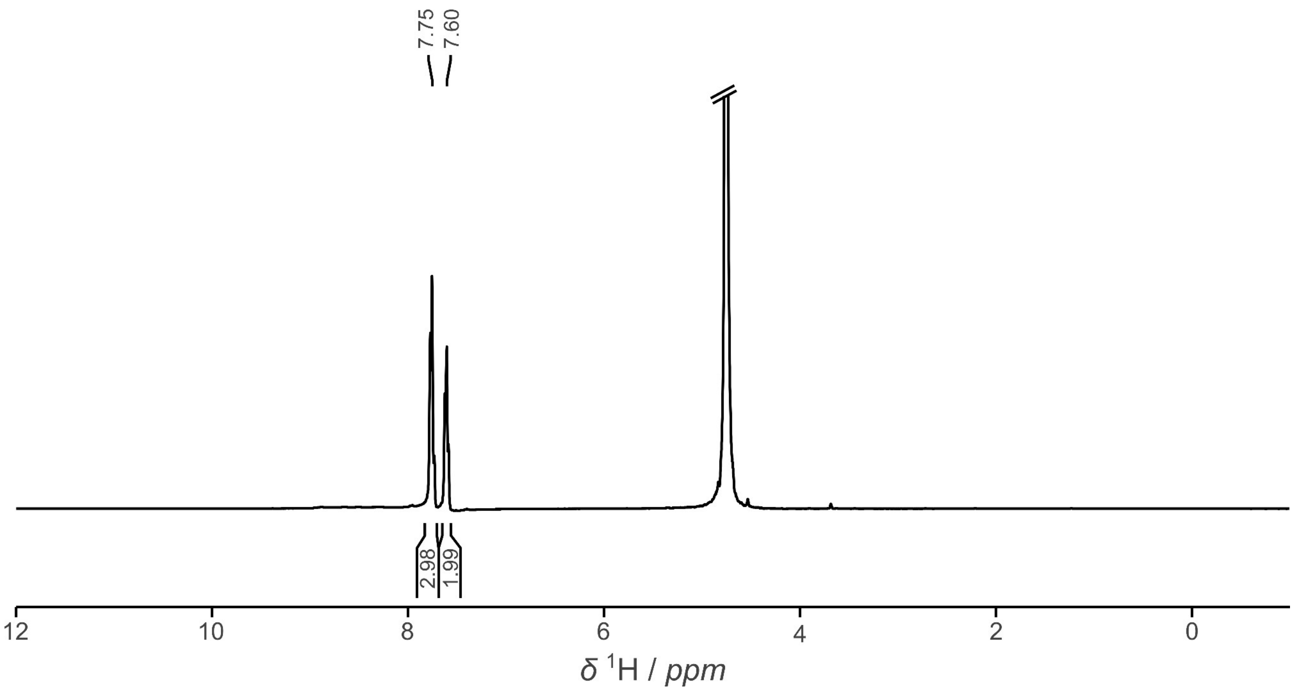

Figure S1. *^{1}H NMR spectrum of ^{15}N benzamidine hydrochloride.*

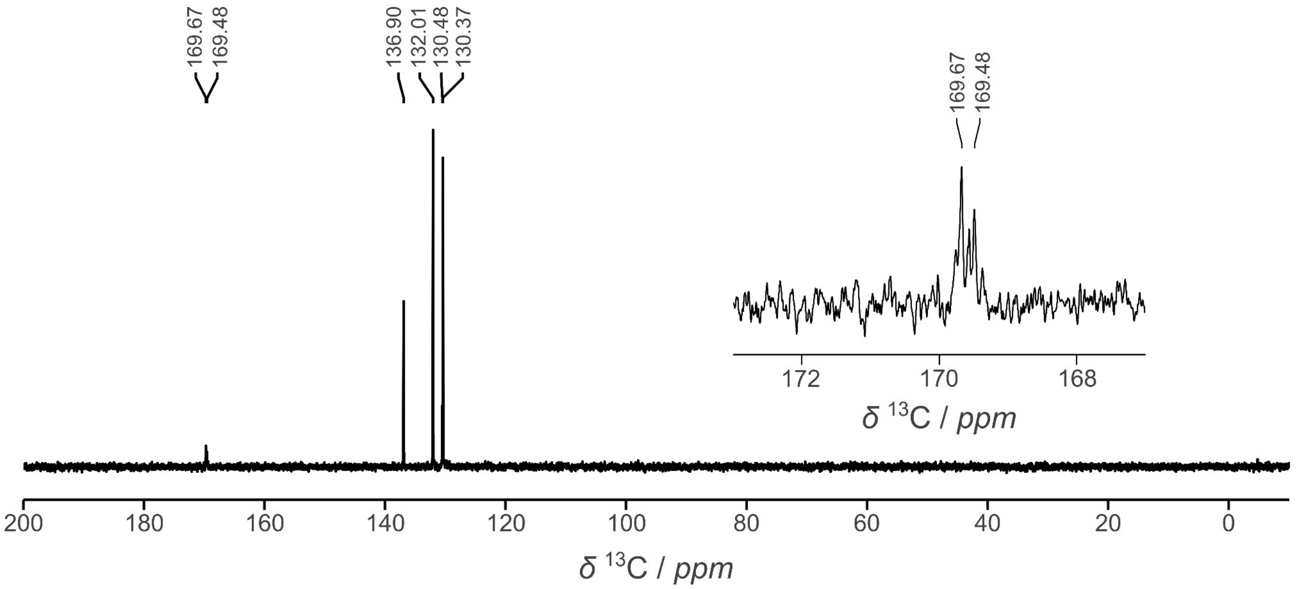

Figure S2. *^{1}H decoupled ^{13}C NMR spectrum of ^{15}N benzamidine hydrochloride. The signal from the amidine carbon is enlarged in the inset.*

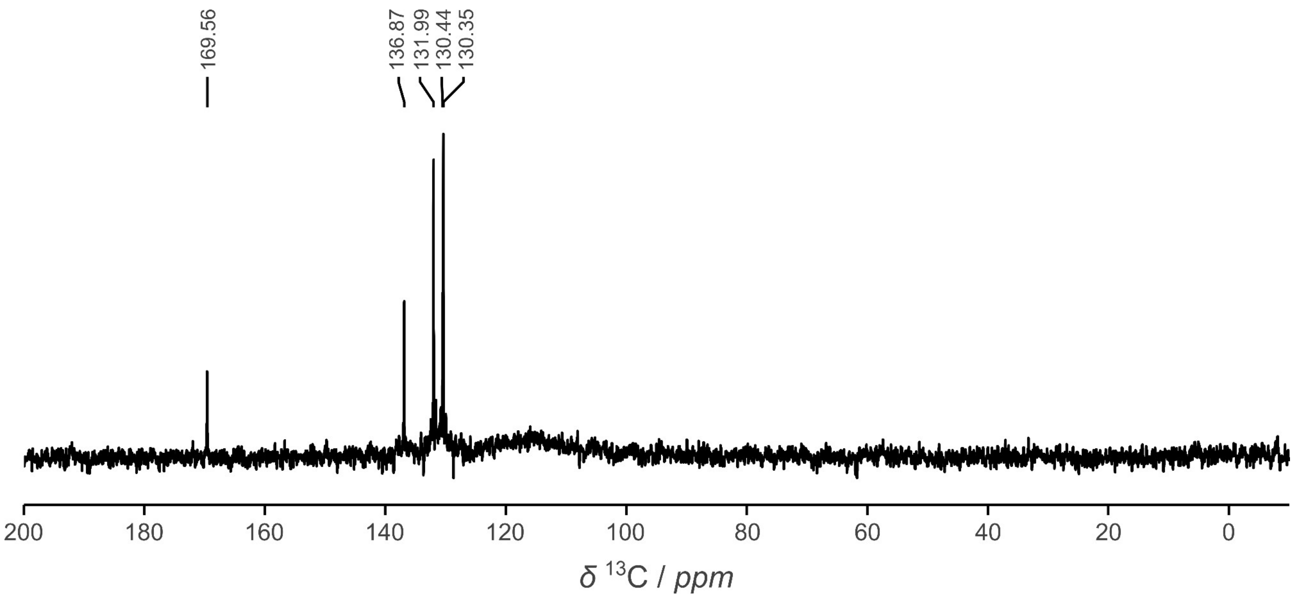

Figure S3. *15N and 1H decoupled 13C NMR spectrum of 15N benzamidine hydrochloride.*

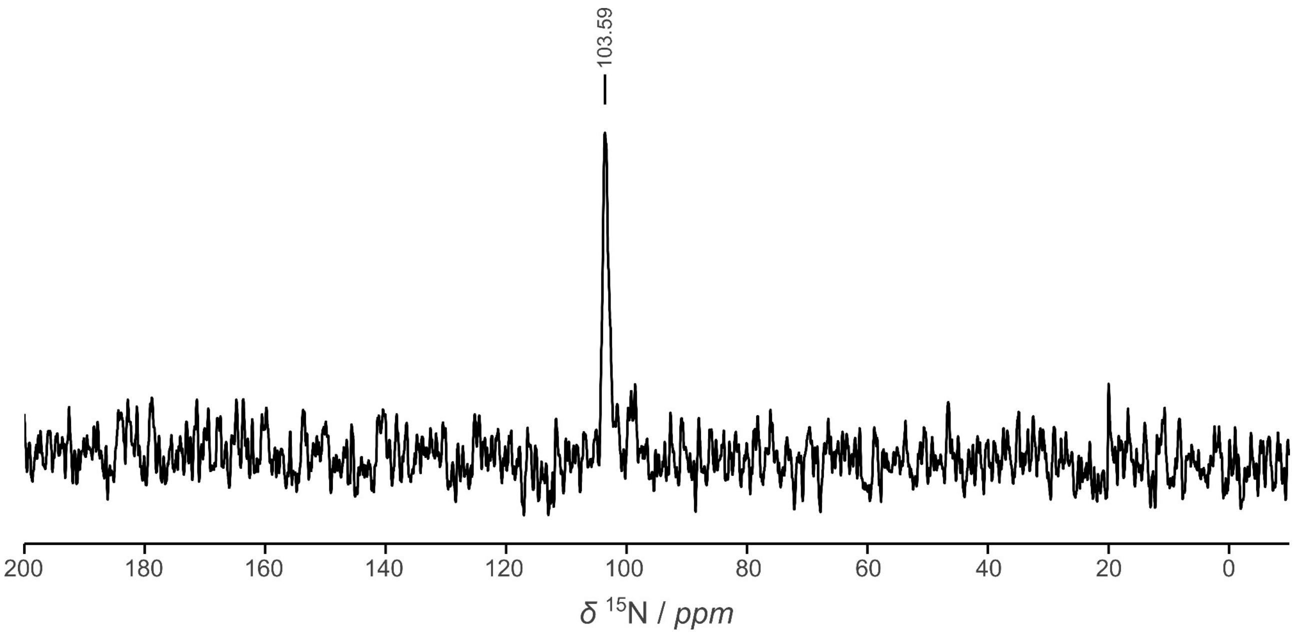

Figure S4. *15N NMR spectrum of 15N benzamidine hydrochloride.*

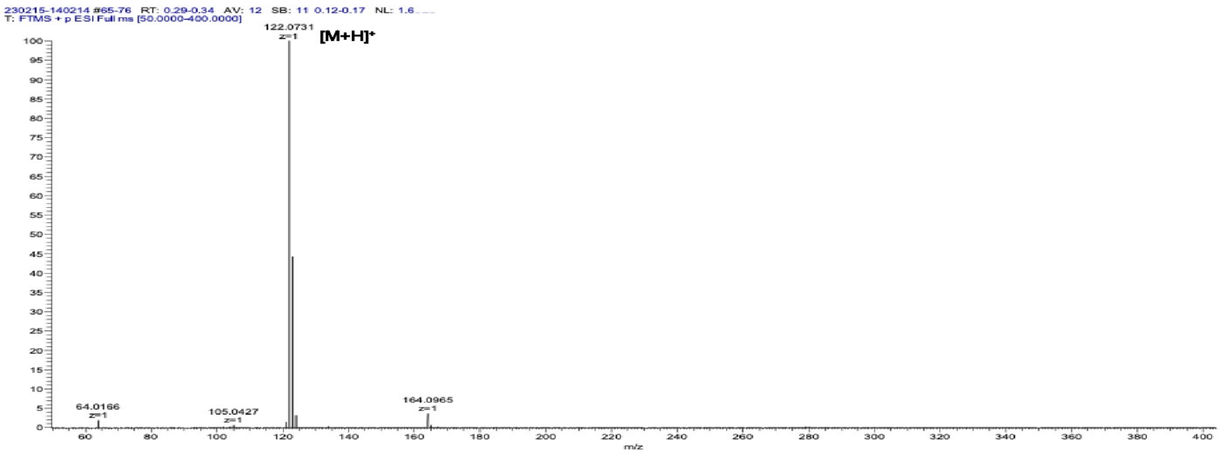

Figure S5. *HRMS spectrum of ^{15}N benzamidine hydrochloride.*

2. Experimental Methods

2.1. Sample Preparation

The stock solution of benzamidine was prepared from synthesized ^{15}N benzamidine in D_2O buffer (10 mM Na_2HPO_4/NaH_2PO_4 buffer or 10 mM tris buffer, pH 7.2). Trypsin protein was also dissolved in D_2O buffer (10 mM tris buffer, pH 7.2). For all of the relaxation experiments with trypsin, the solution was prepared immediately before the experiments.

2.2. Water Hyperpolarization

Water was hyperpolarized similarly to a reported procedure.[2] A 100 µL aliquot of 7:3 (v/v) H_2O/DMSO-d_6 containing 15 mM 4-hydroxy-2,2,6,6-tetramethylpiperidine-1-oxyl (TEMPOL) radical was loaded into a HyperSense DNP polarizer (Oxford Instruments, Abingdon, UK). The sample was irradiated with 100 mW, 94.005 GHz microwaves for 40 minutes at a temperature of 1.4 K. The hyperpolarized sample was then dissolved with 10 bar heated buffer (10 mM sodium phosphate buffer, pH 7.2 for the signal enhancement studies and 10 mM Tris·HCl, pH 7.2 for the relaxation studies with or without trypsin) in D_2O. Finally, a gas driven injector system, with forward and back pressures of 262 psi and 150 psi was used to inject the hyperpolarized water into a 5 mm NMR tube.[3] The injection time was 365 ms. The NMR tube was preloaded in a broadband (BBO) probe in the 400 MHz NMR spectrometer (Bruker Biospin) before the start of the injection.

2.3. NMR Experiments

2.3.1. Non-Hyperpolarized ^{15}N NMR Experiments

The non-hyperpolarized ^{15}N NMR experiment in Fig. 1 was performed with 9.08 mM of benzamidine in Fig. 1. Data was acquired with 400 scans. A delay of 90 s between scans was used. In each scan, a total of 12,174 time-domain data points were acquired with a dwell time of 41.07 µs to measure ^{15}N signals. A Fourier transform, phase correction and baseline correction were applied in Topspin 4.1.4 software (Bruker Biospin).

2.3.2. Non-Hyperpolarized J-CP NMR Experiments

The *J*-CP experiments occurred during a DIPSI-1 spin-locking pulse sequence with a nutation field of 3,125 Hz applied on both the ^{1}H and ^{15}N channels. The duration of the spin lock was 160 ms. The frequency offsets of the pulses were ω_N = 105 ppm and ω_H = 8 ppm. For the *J*-CP enhancement analysis mentioned in Fig. 1, 9.08 mM of benzamidine was used. As above, 12,174 time-domain data points were acquired with a dwell time of 41.07 µs. In the experiment, a 90 s delay between scans were applied and data was acquired in 400 scans. Finally, the free induction delay (FID) was processed as described in earlier sections. The integral was normalized with concentration of benzamidine as well as the number of scans.

2.3.3. HyperW NMR Experiments

Hyperpolarized water was mixed with 50 µL preloaded benzamidine solution in 10 mM sodium phosphate buffer, pH 7.25, in D_2O. For the signal enhancement studies, the preloaded solution was diluted to contain 8.92 ± 0.24 mM, 9.99 ± 0.38 mM and 9.07 ± 0.41 mM benzamidine for the *J*-CP, NOE and INEPT polarization transfer experiments, respectively. The dilution occurred by the injection of the hyperpolarized water solution. The NMR experiments were started with delays of 500 ms, 15 s and 500 ms after injection of hyperpolarized water for *J*-CP, NOE and INEPT respectively. *J*-CP was performed with τ_{CP} = 160 ms and other parameters were as described in the non-hyperpolarized *J*-CP experiments. The NOE experiment was performed by applying a hard pulse on the ^{15}N channel. Refocused INEPT was measured with $\tau_1 = 1/(4J_{HN})$, $\tau_2 = 1/(6J_{HN})$ and J_{HN} = 92 Hz. For all hyperpolarized experiments in Fig. 1, signal acquisition and processing were performed in triplicates as described in the above section. The enhancements of ^{15}N magnetization in benzamidine in hyperpolarized *J*-CP, NOE and INEPT experiments were analyzed by comparing the signal integrals to the integral of the direct ^{15}N signal from a non-hyperpolarized benzamidine sample. Similarly, the signal enhancement in the hyperpolarized *J*-CP experiment was calculated by comparison to the ^{15}N peak integral of non-hyperpolarized *J*-CP experiments. The integrals were normalized with the number of scans as well as the benzamidine concentration to calculate the signal enhancement factors.

Hyperpolarized water mediated *J*-CP induced ^{15}N buildup curves for benzamidine were measured in separate experiments with τ_{CP} = 2 ms, 20 ms, 40 ms, 80 ms, 100 ms, 120 ms, 140 ms and 160 ms. The final concentration of benzamidine was 1.23 ± 0.11 mM. Each set of data was acquired and processed as described above. The integrals were normalized with the concentration of benzamidine. Initial rates of signal buildup were calculated from the slope of the lines connecting the first two data points in the experiment.

The ^{15}N NOE buildup experiment was started with delay of 500 ms after injecting hyperpolarized water. A series of ^{15}N spectra were acquired using 9° flip-angle pulses with delay of 5 s. The benzamidine concentration was 11.77 ± 2.64 mM. The NOE buildup was analyzed by integrating the ^{15}N NMR signal. The integrals were normalized with the benzamidine concentration. Data points were fitted using Eq. S1.[4]

$$\frac{d}{dt}\begin{bmatrix} H_Z(t) \\ N_Z(t) \end{bmatrix} = \begin{bmatrix} -R_1^H & 0 \\ k_{H\to N} & -R_1^N \end{bmatrix} \begin{bmatrix} H_Z(t) \\ N_Z(t) \end{bmatrix} \qquad \text{Eq. S1}$$

Here, R_1^H and R_1^N are the relaxation rates of the respective nuclei, and $k_{H\rightarrow N}$ the overall polarization transfer rate constant from proton to nitrogen nuclei. This equation was solved numerically, step-by-step for each time period between pulses. The signal depletion for each pulse was accounted for by multiplication with a factor of cos(α), α = 9°.[5] The parameters R_1^N, R_1^H and $k_{H\rightarrow N}$ were obtained from fitting the calculated curves to the experimental data using Python and the least_squares function in the Scipy library.

2.3.4. Relaxation Experiments with Protein

A single scan Carr-Purcell-Meiboom-Gill (CPMG) pulse sequence incorporating a *J*-CP element was used to measure ^{15}N R_2 relaxation rates of benzamidine (Fig. S6). The benzamidine concentration was nominally 1 mM in 10 mM Tris buffer, pH 7.25 in the absence and presence of 50 µM trypsin. A DIPSI-1 spin-locking pulse sequence[6] with a nutation field of 3,125 Hz on both ^1H and ^{15}N, with τ_{CP} = 90 ms was used for the experiment. The pulsing delay between two π pulses was 2τ = 6.7 ms in the CPMG portion for the relaxation measurement. In each echo, 512 data points were acquired during the entire delay of 6.7 ms. The ^{15}N spectral width was 39,062 Hz. The acquired data points in each echo were first separated using Python. A sine-shaped window function with the maximum at the center of the echo was applied. The start and end positions of each echo were further extended with zero filling. Finally, a Fourier transform was performed, to obtain a spectrum from each echo and the magnitude of the complex valued signal was calculated. The integrals of the peak from each echo were collected and fitted using scipy.optimize library in Python to an exponential function, $S = a e^{-R_2 t} + b$, to obtain R_2. The average values of R_2 were calculated with error propagation from the fitting error for each data set.

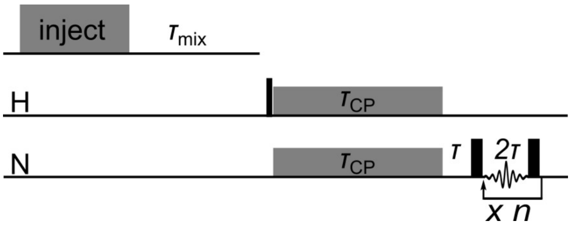

***Figure S6**. J-CP coupled CPMG pulse sequence for R_2 relaxation measurement.*

3. Exchange Rate Calculation

The exchange rate of the amidine protons in benzamidine is estimated according to the base catalyzed mechanism involving OH^- as the catalyst. The second order rate constants for the transfer of the two protons of benzamidine nitrogen were reported as $k = 4.6\cdot10^{10}$ $M^{-1}s^{-1}$ and $5.8\cdot10^{10}$ $M^{-1}s^{-1}$ at 27°C.[7] At pH 7.25, $[OH^-] = 10^{-6.75}$ M, resulting in $k_{ex} = 8.2\cdot10^3$ s^{-1} and $1.0\cdot10^4$ s^{-1} for the two amidine protons.

In the absence of a specific measurement, the rate constant can also be estimated as[8]

$$k_{tr} = k_D \frac{10^{\Delta pK}}{10^{\Delta pK} + 1} \qquad \text{Eq. S2}$$

Here, $k_D \approx 10^{10}$ $M^{-1}s^{-1}$ is the second order rate constant for the diffusion controlled complex formation between an exchangeable amidine proton and the catalyst.[4] ΔpK is the difference of the

acidity constants pK_a of the amidine proton and the conjugate acid of the catalyst. For hydroxide base catalysis, $\Delta pK = 2.44$, considering that the pK_a of benzamidine and water are 11.56 and 14.0, respectively.[9] Using these values, $k \approx 10^{10}\,M^{-1}s^{-1}$ and $k_{ex} \approx 1.7\cdot10^{3}\,s^{-1}$.

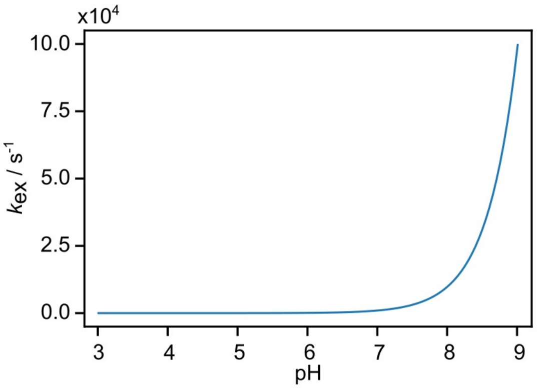

Figure S7. *pH dependence of the exchange rate of amidine protons in benzamidine at 25 °C. The curve was calculated using Eq. S2.*

4. *J*-CP Buildup Curve

Experiments with variable D_2O concentration were performed to assess the influence of deuteration on the CP signal buildup rates (Fig. S8). The cross-polarization experiments were performed as described in section 2.3.2 with spin lock varied between 2 ms to 160 ms. A delay of 80 s between scans was applied to acquire 100 scans. The FID was processed as as described in earlier sections. The integral was normalized with the concentration of benzamidine, as well as the number of scans. The initial rate of signal increase was estimated from the first two data points as the ratio of the integral differences between 2 ms and 20 ms divided by the CP time difference. The ratio of these rates is 1:0.9:0.6 for the 90% *vs*. 50% *vs*. 10% water samples.

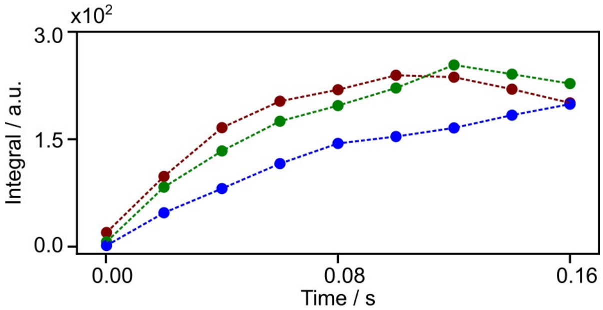

Figure S8. *Effect of water during J-CP magnetization transfer processes with different CP times. The sample consisted of benzamidine in 90% (maroon), 50% (green) and 10% (blue) of water in* D_2O *at pH 7.25. The benzamidine concentration was 26.5 mM, 25.2 mM and 22.3 mM, respectively.*

5. Water T_1 Relaxation Experiments

Water T_1 relaxation experiments were performed with proton concentrations of 90%, 50%, 10% and 3.5% in H_2O/D_2O mixtures. Experiments were conducted first in absence of TEMPOL radial. Relaxation experiments in presence of 0.45 mM TEMPOL further performed in 3.5% water

containing solution to mimic the conditions in the DNP experiments. In the DNP experiments, TEMPOL was used for hyperpolarization and remained in the solution after dissolution and dilution of the sample. Finally, T_1 analysis of previously dissolved DNP samples was carried out without hyperpolarization. Data was acquired using an inversion-recovery pulse sequence with a variable delay of 1 ms to 90 s between π and π/2 pulses. The experiments incorporated pulsed field gradients to alleviate radiation damping, specifically a crusher gradient after the inversion pulse followed by a continuous low-amplitude gradient of 1.36 G/cm during the inversion time. A total of 8192 time-domain data points were acquired with a dwell time of 62.4 μs. A Fourier transform, phase correction and baseline correction were applied in Topspin 4.1.4 software (Bruker Biospin). The integrals of the water peak were calculated and fitted in Python to an exponential function, $I_t = a(1 - b e^{-R_1 t})$.

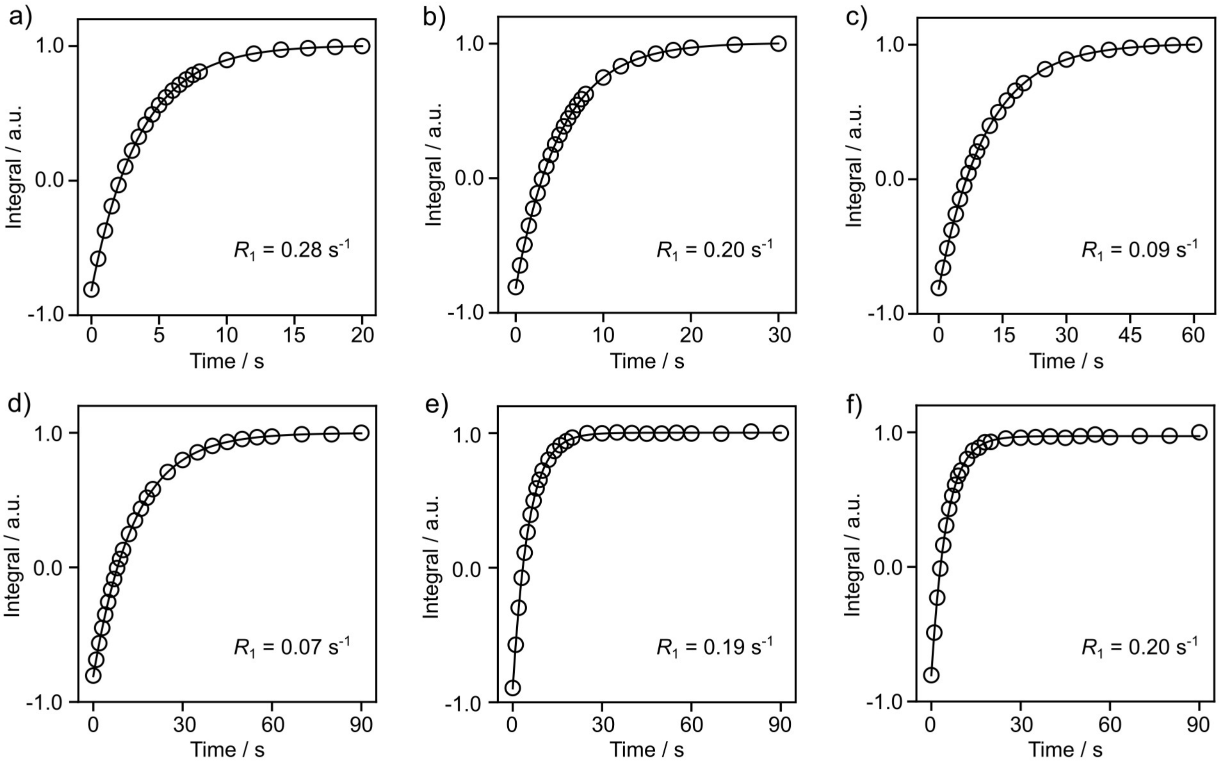

Figure S9. *R_1 relaxation rates of water proton with variable water proton content in H_2O/D_2O mixtures. a) 90% water protons. b) 50% water protons. c) 10% water protons. d) 3.5% water protons. e) 3.5% water protons with 0.45 mM of TEMPOL. f) Relaxation of water protons in a sample from a DNP dissolution measured without hyperpolarization.*

6. Measurement of *J*-CP Efficiency

6.1. Effect of Exchange Rates

The exchange rate dependence of the *J*-CP efficiency was explored using samples of 260 mM benzamidine at different pH values ranging from 6.1 to 8.0. *J*-CP experiments were performed at 25 °C in a single scan, following the above described parameters. The CP time was 100 ms. Acquired data were processed and integrated using Topspin according the described procedures.

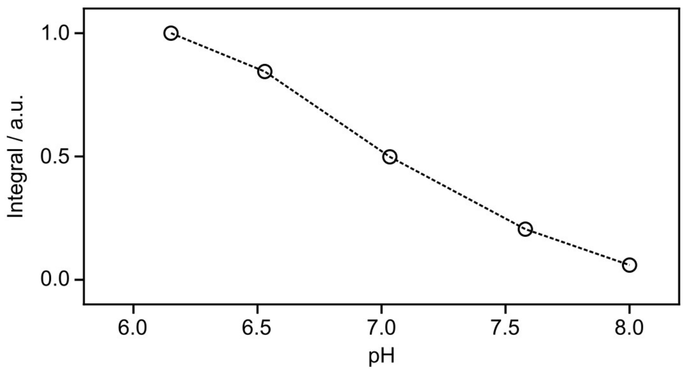

Figure S10. *Integrals of ^{15}N signals of benzamidine at different pH after polarization transfer from water using J-CP. Experiments were performed without hyperpolarization. A sample of 260 mM benzamidine in 90% H_2O/10% D_2O containing buffer was used in this experiment. The data points in the figure are at pH values of 6.15, 6.53, 7.03, 7.58 and 8.00, which represent the amidine proton exchange rates of 140 s^{-1}, 340 s^{-1}, 1050 $s^{-1,}$ 3750 s^{-1} and 10000 s^{-1}, respectively (Fig. S7).*

6.2. Effect of *J*-CP Parameters

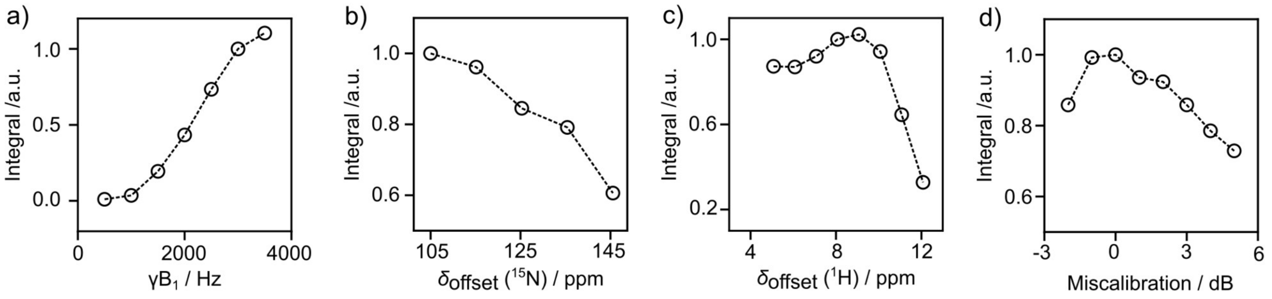

Figure S11. *Dependence of ^{15}N signal integrals of benzamidine at pH 7.25 on J-CP parameters. a) Dependence on nutation field strength γB_1 of the DIPSI-1 sequence. b) Dependence on ^{15}N frequency offset. c) Dependence on ^{1}H frequency offset. d) Effect of miscalibrating the radio-frequency power of the ^{15}N channel. The remaining experimental parameters were as described in the text. The integrals in all panels were normalized to the same scale.*

The efficiency of polarization transfer from water protons to the ^{15}N signal of benzamidine using *J*-CP was investigated as a function of several parameters. Samples of 260 mM benzamidine at pH 7.25 in 90% water containing buffer were used. The *J*-driven cross-polarization experiments were performed using a DIPSI-1 spin-locking pulse sequence. First, the nutation field of spin locking pulse was varied between γB_1=500 Hz to 3500 Hz. Pulse offsets of 105 ppm and 8 ppm were used for ^{15}N and ^{1}H, respectively (Fig. S11a). The γB_1=3125 Hz used in the experiments is near the maximum in the figure and avoids excessive radio-frequency power. The ^{15}N offset dependence was characterized by varying the offset by up to 1600 Hz from the 105 ppm. The nutation field was fixed at γB_1=3125 Hz and the ^{1}H pulse offset was at 8 ppm (Fig. S11b). Similarly, the ^{1}H offset was varied by up to 2920 Hz from 4.7 ppm, while keeping the ^{15}N offset fixed at 105 ppm (Fig. S11c). In the case of ^{15}N, a reduction of approximately 20% in the signal is seen at an offset of 20 ppm. The offset dependence in the case of ^{1}H is more complicated. This is likely due to the presence of two signals, for water and amidine protons, in the spectrum. Furthermore, the effect of miscalculating of ^{15}N power was verified with radio-frequency amplitude changes between -2.5 and 5 dB, while keeping the pulse length constant (Fig. S11d). For all experiments, the CP time was 100

ms and 12,174 time-domain data points were acquired with a dwell time of 41.07 µs. The data was processed and integrated as described above.

6.3. Line Shape and Frequency Difference

The efficiency of the *J*-CP pulse sequence can be rationalized by comparing the line widths in the spectra, and the frequency differences between the peaks to the bandwidth of the DIPSI element. ^{1}H NMR spectra of benzamidine show readily distinguishable signals for the amidine protons near 8.5 ppm at low pH. At the experimental pH of 7.25, these signals become broad and nearly indistinguishable from the baseline due to exchange with the water protons. A simulation of the line shapes using the Bloch-McConnell equations, approximating the system as a two-site exchange problem with k_{ex}=1,700 s^{-1} and a population factor of amidine proton of 0.0002 indicates that the amidine peak, separate from the water peak, has a predicted line with on the order of 500 Hz.

The DIPSI pulse sequence should be chosen with the a radio-frequency field strength to spin-lock the proton spins while avoiding excessive radio frequency power deposition. The sequence with γB_1=3,125 Hz has a decoupling bandwidth of $\pm 0.4\times\gamma B_1$=1,250 Hz.[10] The sequence readily covers the frequency range encompassing the predicted line width of the amidine peak. Additionally, the chemical shift difference between the ^{1}H spin lock offset of 8 ppm and the frequency of the water signal is approximately 1,300 Hz, therefore, protons exchanging with water should also remain at least partially spin-locked.

7. ^{15}N Signal-to-Noise Ratio

The signal-to-noise ratio of the ^{15}N signal from hyperpolarized *J*-CP experiments was calculated as the ratio of the maximum signal intensity and the standard deviation of noise in the 60-70 ppm region. For 9 mM benzamidine, the hyperpolarized *J*-CP experiment (Fig. 1) provided a signal-to-noise ratio (SNR) of 550. Thus, ^{15}N detection of an estimated 50 µM benzamidine can be achieved with a SNR of 3 under the same conditions.

8. NOE Data

Table S1. *Fitting results from NOE buildup curves. The error ranges in the average $k_{H\to N}$, R_1^H and R_1^N are the standard deviation of three experiments.*

Exp. No.	$k_{H\to N}$ / s^{-1}	R_1^H / s^{-1}	R_1^N / s^{-1}	Average $k_{H\to N}$ / s^{-1}	Average R_1^H / s^{-1}	Average R_1^N / s^{-1}
Exp. 1	1.41	0.03	0.13	1.57 ± 0.13	0.02 ± 0.01	0.17 ± 0.06
Exp. 2	1.72	0.02	0.25			
Exp. 3	1.58	0.01	0.12			

9. Protein Interaction Data

Table S2. *^{15}N R_2 Relaxation of benzamidine in the absence and presence of Trypsin. Error ranges in R_2 are from the Jacobian of the fitting procedure. The error ranges in the average R_2 are propageted from R_2 error ranges.*

	[Benzamidine] / mM	[Trypsin] / µM	R_2 / s^{-1}	Average R_2 / s^{-1}
Exp. 1	0.95	-	32.3 ± 4.9	31.3 ± 2.5
Exp. 2	0.87	-	32.3 ± 3.6	
Exp. 3	0.77	-	29.2 ± 4.2	
Exp. 1	0.88	49.3	42.2 ± 5.0	41.1 ± 4.0
Exp. 2	0.89	48.2	42.4 ± 7.6	
Exp. 3	0.77	45.3	38.6 ± 6.9	

10. References

(1) Creary, X.; Sky, A. F. Reaction of Arylbromodiazirines with Azide Ion. Evidence for N-Azidodiazirine Intermediates. *J. Am. Chem. Soc.* **1990**, *112* (1), 368–374.

(2) Hilty, C.; Kurzbach, D.; Frydman, L. Hyperpolarized Water as Universal Sensitivity Booster in Biomolecular NMR. *Nat. Protoc.* **2022**, *17* (7), 1621–1657.

(3) Bowen, S.; Hilty, C. Rapid Sample Injection for Hyperpolarized NMR Spectroscopy. *Phys. Chem. Chem. Phys.* **2010**, *12* (22), 5766–5770.

(4) Harris, T.; Szekely, O.; Frydman, L. On the Potential of Hyperpolarized Water in Biomolecular NMR Studies. *J. Phys. Chem. B* **2014**, *118* (12), 3281–3290.

(5) Kim, J.; Liu, M.; Chen, H.-Y.; Hilty, C. Determination of Intermolecular Interactions Using Polarization Compensated Heteronuclear Overhauser Effect of Hyperpolarized Spins. *Anal. Chem.* **2015**, *87* (21), 10982–10987.

(6) Novakovic, M.; Jayanthi, S.; Lupulescu, A.; Concilio, M. G.; Kim, J.; Columbus, D.; Kuprov, I.; Frydman, L. Heteronuclear Transfers from Labile Protons in Biomolecular NMR: Cross Polarization, Revisited. *J. Magn. Reson.* **2021**, *333*, 107083.

(7) Perrin, C. L.; Schiraldi, D. A.; Lin, G. M. L. Positional Selectivity in an Encounter-Controlled Reaction: Base-Catalyzed Proton Exchange in Amidinium Ions. *J. Am. Chem. Soc.* **1982**, *104* (1), 196–201.

(8) Eigen, M. Proton Transfer, Acid-Base Catalysis, and Enzymatic Hydrolysis. Part I: ELEMENTARY PROCESSES. *Angew. Chem. Int. Ed.* **1964**, *3* (1), 1–19.

(9) Englander, S. W.; Downer, N. W.; Teitelbaum, H. Hydrogen Exchange. *Annu. Rev. Biochem.* **1972**, *41* (1), 903–924.

(10) Shaka, A. J.; Lee, C. J.; Pines, A. Iterative Schemes for Bilinear Operators; Application to Spin Decoupling. *J. Magn. Reson. (1969)* **1988**, *77* (2), 274–293.
